# Beyond Pain Scores: The Emerging Era of Pain Biomarkers

**DOI:** 10.3390/biomedicines14102229

**Published:** 2026-10-02

**Authors:** Anders O. Larsson, Mats B. Eriksson

**Affiliations:** 1 Department of Medical Sciences, Section of Clinical Chemistry, Uppsala University, 751 85 Uppsala, Sweden; anders.larsson@akademiska.se; 2 Department of Surgical Sciences, Section of Anaesthesiology and Intensive Care Medicine, Uppsala University, 751 85 Uppsala, Sweden; 3 NOVA Medical School, New University of Lisbon, 1099-085 Lisbon, Portugal

Pain is a vital protective mechanism that helps us avoid acute injuries and, ultimately, their potentially disabling consequences. However, pain is a double-edged sword, as it can become a major source of suffering and functional limitation.

Beyond its profound impact on patient quality of life, chronic pain imposes a considerable socioeconomic burden through increased healthcare utilization and loss of productivity, with recent estimates suggesting costs corresponding to approximately 4% of gross domestic product and disability levels that rank among the highest worldwide [1].

Pain remains one of the most prevalent causes of healthcare utilization worldwide, yet it is also one of medicine’s most difficult symptoms to quantify objectively. Although advances in molecular biology, neuroimaging, systems neuroscience, and computational medicine have improved our understanding of pain mechanisms, clinicians still rely primarily on patient self-report to assess pain intensity and treatment response. The search for pain biomarkers therefore represents one of the most important translational challenges in contemporary pain medicine.

The concept of a pain biomarker is not new. Biomarkers have long been defined as objectively measurable characteristics that indicate normal biological processes, pathological processes, or responses to therapeutic interventions [2]. Pain, however, presents unique challenges because it cannot be equated with nociceptive input alone. Rather, pain is a multidimensional experience shaped by interactions among peripheral signaling, central neural processing, emotions, cognition, memory, expectations, genetics, and environmental influences.

This complexity was acknowledged when the International Association for the Study of Pain revised its definition of pain, emphasizing that pain is both a sensory and emotional experience [3]. Consequently, the search for biomarkers has evolved from a quest for a single objective measure toward the identification of multidimensional biological signatures that reflect specific mechanisms and clinical phenotypes.

A major conceptual advance has been the recognition that chronic pain is mechanistically heterogeneous. Nociceptive, neuropathic, and nociplastic pain (i.e., pain due to changeable alterations in nociceptive processing) represent distinct but sometimes overlapping processes [4]. This concept has encouraged researchers to seek mechanism-specific biomarkers rather than a universal biomarker applicable to all pain conditions.

Among the earliest and most extensively studied biomarker candidates are inflammatory mediators. Neuroimmune interactions are now recognized as critical contributors to persistent pain states [5]. In parallel, astrocytes and glial cells have emerged as active participants in pain generation and maintenance through the regulation of central sensitization, neuroinflammation, and synaptic plasticity [6].

Numerous studies have reported altered concentrations of cytokines, chemokines, and growth factors in patients with chronic pain. However, despite promising findings, no individual inflammatory mediator has demonstrated sufficient sensitivity and specificity to be adopted as a standalone clinical biomarker. A systematic review of cerebrospinal fluid cytokines concluded that although inflammatory alterations are consistently observed, considerable methodological heterogeneity remains [7]. Consequently, inflammatory markers are increasingly viewed as components within broader biomarker panels rather than independent diagnostic tests.

In parallel, neuroimaging has provided some of the strongest evidence that objective correlates of pain can be identified in the human nervous system. Functional and structural studies have demonstrated widespread alterations involving sensory, emotional, motivational, and cognitive networks [8]. Recognizing the limitations of single modalities, several authors have advocated for the development of composite biomarker signatures integrating imaging, physiological, and molecular data [9].

A major milestone was reached when Wager et al. developed a functional MRI-based neurological pain signature capable of predicting experimentally induced pain intensity with high accuracy [10]. Although neuroimaging biomarkers remain technically demanding and expensive to apply, they have substantially advanced understanding of pain-related brain mechanisms.

The clinical implementation of neuroimaging biomarkers nevertheless raises important methodological, legal, and ethical questions. These challenges were comprehensively discussed by Davis et al., who emphasized the scientific opportunities and the limitations of using brain imaging as diagnostic evidence in chronic pain [11].

Recent work suggests that future advances will emerge from multimodal approaches. A contemporary overview highlights the growing role of artificial intelligence, machine learning, multimodal integration, and precision medicine in biomarker development [12].

The rapid emergence of omics technologies has further transformed the field. Epigenetic modifications have increasingly been implicated in chronic pain pathophysiology and may contribute to individual susceptibility, persistence of symptoms, and treatment responses [13]. Likewise, proteomic investigations have revealed systemic alterations involving inflammatory, metabolic, and stress response pathways in individuals with widespread pain syndromes [14,15,16].

The potential of omics-driven precision medicine is further highlighted by studies seeking objective molecular signatures capable of increasing diagnostic accuracy and guiding individualized treatment strategies [17].

One contribution demonstrates that cerebrospinal fluid metabolomics could identify ongoing analgesic medication use in patients with neuropathic pain, illustrating how metabolomic profiling can reveal biological information not readily accessible through conventional assessment [18].

Another study investigated platelet membrane proteins as candidate biomarkers of pain in patients with severe dementia, a patient group in whom communication barriers often complicate pain assessment [19]. The identification of pain biomarkers could lead to adjustment of the therapeutic strategy, contributing to faster and more adequate control of pain and reductions in patient-associated suffering.

Similarly, Karpin et al. demonstrated that patterns of central sensitization and psychological characteristics could distinguish complex regional pain syndrome from other chronic pain conditions [20]. These findings underline the importance of integrating biological and phenotypic dimensions when developing clinically useful biomarkers.

Neuroimaging perspectives were provided through experimental work using FDG-PET to characterize activation within the pain matrix in a neuropathic pain model [21]. Such studies provide additional support for the concept that objective neural signatures of pain-related processing can be identified and quantified.

Taken together, these contributions reflect a central theme emerging throughout pain biomarker research: the increasing shift away from single-marker approaches toward multidimensional and systems-based strategies. The most promising biomarkers will likely combine molecular signatures, neuroimaging findings, sensory testing, physiological measurements, and clinical phenotyping.

The transition from acute to chronic pain remains one of the most important targets for biomarker discovery. Understanding who develops persistent pain after injury, surgery, or disease could fundamentally alter preventive strategies. Neuroimaging studies continue to provide valuable insights into pain-related brain plasticity and disease progression [22]. Simultaneously, evidence increasingly suggests that long-term pain states are accompanied by structural and functional reorganization throughout the nervous system [23].

Despite substantial progress, important challenges remain. Reproducibility, external validation, standardization of methodologies, and biological heterogeneity continue to limit the clinical translation of research findings. Moreover, pain remains inherently subjective. Biomarkers are unlikely to replace patient-reported outcomes; rather, their value may lie in characterizing mechanisms, identifying treatment responders, improving prognostication, and supporting precision pain medicine.

In conclusion, the field of pain biomarkers has considerably matured in the past decade. Established discoveries involving neuroimmune pathways, neuroimaging, and omics technologies have laid a strong scientific foundation, and recent work has expanded the vision toward integrated, multimodal biomarker signatures. Although no single biomarker has yet achieved routine clinical implementation, the convergence of molecular biology, neuroscience, computational medicine, and artificial intelligence suggests that clinically actionable biomarker signatures will soon become a component of personalized pain management.
